# LOWER TRAPEZIUS TRANSFER FOR IRREPARABLE ROTATOR CUFF TEAR: SYSTEMATIC REVIEW

**DOI:** 10.1590/1413-785220253302e286068

**Published:** 2025-06-02

**Authors:** Guilherme Trigueiro Alarcon, Jyotis Natacha Brito Corbin, Eduardo Misao Nishimura, Luciano Pascarelli, Eiffel Tsuyosho Dobashi

**Affiliations:** 1 Sociedade Brasileira de Ortopedia e Traumatologia (SBOT), Sao Paulo, SP, Brazil.; 2 Hospital IFOR, Rede D’Or São Luiz, Grupo de Ombro, Sao Paulo, Brazil.

**Keywords:** Shoulder, Rotator Cuff Tear, Shoulder Injuries, Systematic Review, Ombro, Ruptura do Manguito Rotador, Lesões do Ombro, Revisão Sistemática

## Abstract

To evaluate functional results considering the final stages of function and postoperative pain in patients with irreparable rotator cuff tears undergoing surgical treatment by lower trapezius transfer. A systematic review of the literature was carried out following the PRISMA guidelines without restrictions on language and year of publication. The search was carried out in the following databases: MEDLINE/PubMed, EMBASE, Scopus and Cochrane Central Register of Controlled Trials. The main descriptors used were: "Rotator Cuff" OR "Rotator Cuff Injuries" OR Rotator Cuff Tear. The stages analyzed were pain, range of motion and function. We found 215 articles, of which 12 were included involving 374 participants. There was a statistically significant improvement comparing the pre and postoperative periods. The average intensity of pain was 7.1 and decreased to 2.4 according to the VAS. Using the ASES system, the result certainly improved, where the initial average was 49.5 (bad result) and the final average was 78.3 (good result). The results suggest that lower trapezius transfer is effective as a therapeutic option in patients treated by the surgical technique studied, according to the results found. ***Level of Evidence III; Systematic Review.***

## RESUMO

Avaliar os resultados funcionais de pacientes com rupturas irreparáveis do manguito rotador submetidos à transferência do trapézio inferior, considerando dor e função pós-operatória. Realizamos uma revisão sistemática seguindo as diretrizes PRISMA, sem restrições de idioma ou ano de publicação. As buscas foram feitas nas bases MEDLINE/PubMed, EMBASE, Scopus e Cochrane. Utilizamos os descritores: "Rotator Cuff" OR "Rotator Cuff Injuries" OR "Rotator Cuff Tear". Os desfechos analisados foram dor, amplitude de movimento e função. Foram identificados 215 artigos, dos quais 12 atenderam aos critérios de inclusão, totalizando 374 participantes. Houve melhora estatisticamente significativa entre os períodos pré e pós-operatório. A intensidade média da dor, medida pela Escala Visual Analógica (EVA), reduziu de 7,1 para 2,4. Pelo sistema American Shoulder and Elbow Score (ASES), a pontuação aumentou de 49,5 (ruim) para 78,3 (bom). A transferência do trapézio inferior demonstrou eficácia na melhora da dor e função dos pacientes estudados. A análise estatística evidenciou diferença significativa nos desfechos pós-operatórios. ***Nível de Evidência: III, Revisão Sistemática.***

## INTRODUCTION

The topic involving the management of massive and irreparable rotator cuff tears is considered challenging, as surgical outcomes have not yet been definitively clarified. Some authors note a high failure rate in evaluating results.^1,2^ However, in clinical practice, the therapeutic indication is based on the following criteria, such as the rupture of two or more tendons, the duration of symptoms being longer than 6 months, tendon retraction greater than 5 cm, and a fatty infiltration grade of 3 according to the Goutallier classification, to indicate surgical intervention.^3^

In irreparable tears of the subscapularis, supraspinatus, and infraspinatus muscles, patients present significant dysfunction and pain. Thus, functional restoration and pain reduction are considered paramount,^4^ particularly in patients who are not candidates for shoulder arthroplasty due to age or high-level activity. Therefore, tendon transfers are considered surgical options with superior effects when performing reconstruction of the deficient rotator cuff.^2,5^ Recently, the orthopedic literature has highlighted that the lower trapezius transfer (LTT) warrants attention in the treatment of posterosuperior rotator cuff tears, particularly when associated with weakness in external rotation and signs of relapse in external rotation.^6^ Therefore, researchers have shown increasing interest in investigating the effects of this transfer to treat deficiencies in degenerative shoulder conditions.^7^ We know that, initially, LTTs were indicated to restore external rotation surgically in the treatment of brachial plexus paralysis.

A recent systematic review reported that LTT improves clinical outcomes in patients with irreparable rotator cuff tears. It presents a similar complication and reoperation rate when compared with other surgical alternatives in this group of patients.^7,8^ What motivated the authors of this research was the absence of secondary studies evaluating the functional outcomes of surgical treatment. Therefore, this study aims to evaluate the functional results of LTT, considering functional aspects and pain in the pre and postoperative periods of patients with irreparable rotator cuff tears.

## MATERIALS AND METHODS

This systematic review was conducted according to the guidelines of the Preferred Reporting Items for Systematic Reviews and Meta-Analyses – PRISMA.^9^

### Data Sources and Searches

To assist in the search for scientific publications of intervention studies, a clinical question was formulated based on the strategy defined by the PICO acronym.^10^ Therefore, we determined that: P = patients with irreparable rotator cuff injuries; I = interventions with the lower trapezius transfer; C = intervention comparing preoperative and postoperative results; O: the outcomes analyzed were pain and function by the ASES system in the pre and postoperative periods.

For pain assessment, we considered studies that used the Visual Analog Scale (VAS).^11^ For shoulder range of motion measurements, we considered studies that used a goniometer, and for shoulder function assessment, we considered studies that used the following questionnaires: Constant-Murley Score,^12^ American Shoulder and Elbow Surgeons (ASES)^13^ or Quick Disabilities of Arm, Shoulder & Hand (Quick DASH score).^14^

A researcher formulated the electronic search strategy in the databases MEDLINE/PubMed, EMBASE, Scopus, and Cochrane Central Register of Controlled Trials. Additionally, additional articles that could be included were searched in the reference lists of eligible studies.

The search terms used in the database searches were combined with the Boolean operators "AND" and "OR". The terms for the searches were as follows: Arthroplasty; Coracohumeral Impingement; Coracohumeral Impingement Syndrome; Coracohumeral Impingement Syndromes; Cuff Injury; Glenoid Labral Tear; Glenoid Labral Tears; Glenoid, Rotator Cuff Injury; Injuries; Injury; Orthopedic Procedures; Replacement, Shoulder; Rotator; Rotator Cuff; Rotator Cuff Injuries; Rotator Cuff Tear; Rotator Cuff Tear Arthropathy; Rotator Cuff Tears; Rotator Cuff Tendinitides; Rotator Cuff Tendinitis; Rotator Cuff Tendinosis; Rotator Cuff Tendinosis; Rotator Cuff, Coracohumeral Impingement; Rotator Cuff, Labral Tear; Shoulder; Shoulder Fractures; Shoulder Impingement Syndrome; Shoulder Injuries; Shoulder Joint; Shoulder Pain; Shoulder Prosthesis; Tear, Glenoid Labral; Tear, Rotator Cuff; Tears, Glenoid Labral; Tears, Rotator Cuff; Tendinitis, Rotator Cuff; Tendinosis, Rotator Cuff; Tendinosis; Tendinosis, Rotator Cuff.

The inclusion criteria for the studies were: (1) studies with individuals who underwent LTT for irreparable rotator cuff injury; (2) individuals of both sexes; (3) studies in any language; (4) without restrictions regarding the year of publication. The criteria for exclusion of articles were: (1) studies conducted on cadavers; (2) publication in the form of conference abstract, letter, editorial, case report; (3) literature reviews; (4) systematic reviews; (5) LTT for brachial plexus injury; (6) studies comparing LTT with other techniques.

The screening of articles was conducted using the Rayyan software, which enables a rapid selection of eligible studies.^15^ The evaluations of titles, abstracts, and the full reading of the articles were conducted by two researchers independently. Any discrepancies in the identification of articles were resolved among the research team members. After the full reading of the old ones, the following information was collected: authors and year of publication, study design, follow-up time, country where the study was conducted, sample size, average ages, intervention, and outcome results (pain, range of motion, and function).

The electronic search was conducted between January and February 2024.

### Quality assessment of the studies

The analysis was performed by scoring the Methodological Index for Non-Randomized Studies (MINORS) score, which comprises eight items for non-comparative studies and 4 additional items for comparative studies.^16^

### Compliance with Ethical Guidelines

This original article was based on previously published studies; therefore, it did not involve the direct extraction of data from study participants.

## RESULTS

**Figure 1:**
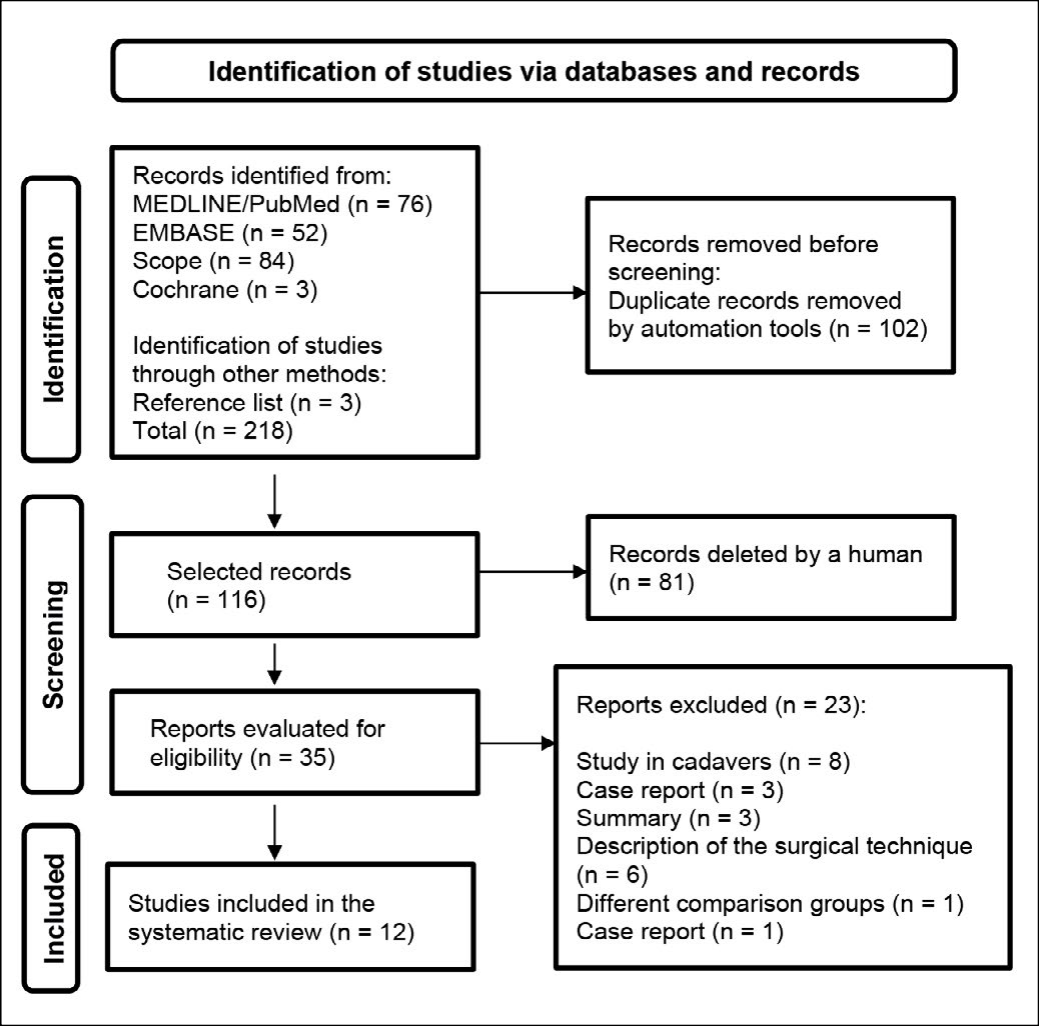
PRISMA flowchart of the study selection process.

A total of 215 articles were identified in the databases used in this study, and three additional articles were selected through searches of the references of the included studies. 102 duplicate articles and 81 were excluded through the screening of titles and abstracts. Thus, we conducted a full reading of 35 articles, of which 12 were included in the qualitative synthesis.^17–28^ (Figure 1 illustrates the flowchart for selecting studies).

Ten studies were classified as retrospective case series,^17,18,20–24,26–28^, one retrospective cohort study,^25^ and one study using a retrospective crossover design.^19^ These studies were conducted in Brazil,^17^ South Korea,^18–20^ Turkey,^21^ United States^22–26,28^ and France.^27^ The twelve studies included a total of 374 participants, with an average age of 61 years (ranging from 40 to 73 years). The participant demographics were as follows: 241 (64.4%) were male, and 133 (35.6%) were female. The average postoperative follow-up time was 22 months, ranging from 6 to 70 months.

The main indications for surgery were persistent pain, failure of conservative treatment,^17–20,22^ massive rotator cuff tear identified on MRI, association with fatty infiltration (Goutallier grade III or IV),^23–25,28^ presence of intact subscapularis^28^ and teres minor tendons, and limitation of active range of motion.^17–20,22^ Other criteria found in the studies were: arthritic changes by Hamada criteria grade 0, 1, 2;^19,28^ patients without neurological diseases;^19^ absence of glenohumeral osteoarthritis on X-rays (Hamada <3); no limitation of passive range of joint movements;^21^ and patients with deltoid muscle paralysis.^25^

The average MINORS score was 11.5 (range from 10 to 12) for non-comparative studies and 18 (range from 17 to 19) for the included comparative studies (Table 1).

**Table 1:** Characteristics of the included studies on lower trapezius transfer for rotator cuff injuries.

Author/ Year/ Country	Study design/follow-up	Sample size	Average age	Type of injury	Intervention	Comparison group	Type of graft	Instruments	MINORS core
Almeida et al., 2023^17^ Brazil	Retrospective case series Pre-operative, 6 and 12 months post-operative.	10 10 Men	40 and 62 years (mean 51.90)	Irreparable rotator cuff tears	Transfer of the lower trapezius	Pre and Post-operative	Knee flexor tendon grafts (semitendinosus and gracilis)	VAS ROM	16/12
Baek et al., 2023^18^ Republic of Korea	Retrospective case series 58.2±5.3 months (48-70 months)	36 24 Men 12 Women	63.4±5.4 years	Irreparable postero-superior rotator cuff tears in the wrist	Lower trapezius transfer assisted by arthroscopy	Pre and Post-operative	Achilles tendon	VAS ROM	16/12
Baek et al., 2022^19^ Republic of Korea	Cross-sectional retrospective study 2 years	42 27 Men 15 Women	63.3±5.8 years	Irreparable postero-superior rotator cuff tears in the wrist	Lower trapezius transfer	Pre and Post-operative	Achilles tendon	VAS ROM	18/24
Baek et al., 2022^20^ Republic of Korea	Retrospective case series 2 years 26-49 months	36 22 Men 14 Women	63.1±65.5 years	Irreparable postero-superior rotator cuff tears	Lower Trapezius Transfer	Pre and Post-operative	Achilles tendon	VAS ROM	19/24
Bozoğlan al., 2023^21^ Turkey	Retrospective case series 29±3 months (24-39)	16 12 Men 4 Women	62±9 years (42-73 years)	Large irreparable rotator cuff tears	Lower trapezius tendon transfer	Pre and Post-operative	Long fibular allograft combined with interpositional repair with fascia lata	VAS ROM	16/12
Chopra et al., 2023 EUA	Retrospective case series 14.6 (range 6 to 45) months	19 17 Men 2 Women	56.7 years (range 29 to 72 years)	Large irreparable rotator cuff tears	Lower trapezius tendon transfer	Pre and Post-operative	Achilles tendon	VAS ROM	16/12
Marigi et al., 2023^22^ EUA	Retrospective cohort comparison 2.9 ± 1.3 years (range 1.0 to 6.3 years)	104 58 Homens 14 Women	56.8 ± 7.9 years	Irreparable rotator cuff tears	Assisted by Arthroscopy Trapezius transfer	Pre and Post-operative	Achilles tendon	VAS ROM Quick DASH score	17/24
Elhassan et al., 2020^23^ EUA	Retrospective case series 14 months follow-up	41 30 Men 11 Women	52 years (range 37-71 years)	Massive and irreparable postero-superior rotator cuff tear	Transfer of the lower trapezius	Pre and Post-operative	Achilles tendon	(1) VAS (2) ROM (3) Quick DASH score	16/10
Stone et al., 2020^24^ EUA	Retrospective case series 1 year follow-up	15 14 Men 1 Women	52 years (age range 31-62 years)	Irreparable posterior-superior rotator cuff tears	Lower trapezius tendon transfer	Pre and Post-operative	Achilles tendon	VAS ROM	16/11
Woodmass et al., 2020^25^ EUA	Retrospective case series The average follow-up was 22±10 months	8 6 Men 2 Women	53 years	Chronic massive posterior-superior rotator cuff tear	Arthroscopy-assisted lower trapezius tendon transfer	Pre and Post-operative	Achilles tendon	VAS ROM ASES function	18/24
Valenti e Werthel et al., 2018^26^ France	Retrospective case series 1 year follow-up	14 8 Men 6 Women	62 years (age range 50 to 70 years)	Irreparable posterior-superior rotator cuff tear	Transfer of the lower trapezius	Pre and Post-operative	Semitendinosus tendon	VAS ROM Constant-Murley score	16/12
Elhassan et al., 2016^27^ EUA	Retrospective case series 47 months follow-up	33 27 Homens 6 Women	53 years (variation from 31 to 66 years)	Massive and irreparable postero-superior rotator cuff tear	Transfer of the lower trapezius	Pre and Post-operative	Achilles tendon	(1) VAS (2) ROM (3) Quick DASH score	16/11

Visual Analog Scale (VAS); Range of motion (ROM); Quick Disabilities of the Arm, Shoulder, and Hand (Quick DASH) score; MINORS score: Methodological Index for Non-Randomized Studies.

Ten studies assessed pain using the EVA scale in the pre and post-operative periods.^17–23,25,27,28^ These studies indicated that there was a significant reduction in pain after surgical intervention. After evaluating the results, we observed a statistically significant improvement when comparing the pre and post-operative periods. The average pain intensity was 7.1 and decreased to 2.4 according to the EVA. Using the ASES system, the result improved positively where the initial average was 49.5 (poor result) and final was 78.3 (good result).

Table 2 summarizes the pre- and post-operative results, considering pain, ROM, and function, as assessed by ASES scales,^17–20,22,28^ the Constant-Murley Score,^21,27^ and the Quick DASH score.^23–25^ There was a statistically significant improvement during outpatient follow-up (p < 0.05).

**Table 2:** Results of the included studies on lower trapezius transfer for rotator cuff injuries.

Study	Pre-operative			Post-operative		
	Pain	Range of motion	Function	Pain	Range of motion	Function
Almeida et al., 2023^17^	7,9	Mean lateral rotation 31° Flexion 84° Abduction 76°	ASES function 26.63±7.34	2.5 (p < 0.001)	Mean lateral rotation improved by 51° Flexion 122° Abduction 101° (p < 0.001)	ASES function 75.24±3.59 (p < 0.001)
Baek et al., 2023^18^	4,5±1,2	Forward elevation 134°±42° Abduction 116°±41° External rotation at 90° Abduction 48°±20° Lateral external rotation 25°±13° Internal rotation at the back 6.3°±1.6°	ASES function 57.1±13.4	1.4±0.7 (p < 0.001)	Frontal elevation 160°±30° (p=0.007) Abduction 149°±32° (p<0.001 *) External rotation at 90° Abduction 69°±17° (p<0.001) Lateral external rotation 43°±12° (p<0.001) Internal rotation on the back 6.5°±1.2° (p=0.283)	ASES Function 83.7 ± 8.2 (p <0.001)
Baek et al., 2022^19^	4,3 ± 1,6	Active frontal elevation 125,4°±34,9° Active external rotation with 0° abduction 22,3°±10,9°	ASES Function 55.8±16.7	1.7 ± 1.3 (p < 0.001)	Active frontal elevation 160.1°±19.5° (p < 0.001) Active external rotation with 0° abduction 54.8°±9.8° (p < 0.001)	ASES function 82.7 ± 8.6 (p < 0.001)
Baek et al., 2022^20^	4,5±61,8	Active frontal elevation 134,2°±653,6° Active external rotation 27,5°±614,3 °	ASES function 47.2±617.3	1.3±61.0 (p < 0.001)	Active front elevation. 165.7°±22.3° (p=0.003) Active external rotation 51.7°±10.9° (p=0.003)	ASES function 84,8±67,6 (p<0.001)
Bozoğlan et al., 2023^21^	6,1±1,1	Active forward flexion 109°±24.7° Active abduction 60°±16.3° Active external rotation 12°±16.9°	Constant - Murley score 24.00 ± 9.43	2.4±1.35 (p=0.007)	Active flexion advance 144°±22.21° in the postoperative period (p=0.005) Active abduction 135°±16.3° (p=0.005) Active external rotation 35°±14.3° (p=0.005)	Constant - Murley score 50.20 ± 14.28 (p=0.008)
Chopra et al., 2023	5,9±2	Elevation 139,5° ±26° External rotation 10,5° ±17° External rotation force 2,8/5° ±1°	ASES function 44.6 ± 18	1.8±2 (p<0.001)	Elevation 147.4°±29° (p=0.31) External rotation 40.5°±13° (p<0.001) External rotation force 4.7/5°±0.5° (p<0.001)	ASES function 71.2 ± 24 (p<0.001)
Marigi et al., 2023^22^	6,2±2,5	Front lift strength 4,0°±0,6° External rotation strength 3,4°±0,5° Internal rotation strength 4,3 °± 0,7 °	Quick DASH score 50.3 ± 14.0	1,1±2,0 (p < 0.00)	Front lift strength 4.5°±0.5° (p<0.001) External rotation strength 4.5°±0.5° (p<0.001) Internal rotation strength 4.8°±0.4° (p=0.005)	Quick DASH score 13,2±15,4 (p = <0.001)
Elhassan et al., 2020^23^	6	Forward flexion 67° External rotation 25°	Quick DASH score 49	2 (p<0.001)	Forward flexion 133° (p<0.001) External rotation 47° (p<0.001)	Quick DASH score 18 (p<0.001)
Stone et al., 202024	NR	Active elevation 98° Abduction 74° External rotation 23°	NR	NR (p < 0.05)	Active elevation 144° (P < 0.0001) Abduction 127° (P < 0.0001) External rotation 43° (P < 0.0001)	NR
Woodmass et al., 2020^25^	2,85	Forward flexion 101° External rotation 33°	ASES function 12.5	1.17 (p = 0.089)	Forward flexion 146° (p = 0.031) External rotation 44° (p = 0.27)	ASES function 24.4 (p = 0.0092)
Valenti e Werthel et al., 2018^26^	7	Flexion of 150° External rotation −20° External rotation with the arm at 90° abduction −10°	Constant - Murley score 35 (range) 20–50)	2 (p<0.001)	Flexion 160° External rotation 24° External rotation with the arm at 90° abduction improved from 40°	Constant-Murley score 60±9 (p<0.001)
Elhassan et al., 2016^27^	NR	Flexion of 70° Abduction of 40° External rotation of 20°	Quick DASH score 52±19	NR	Flexion of 120° Abduction of 90° External rotation of 50° (p < 0.01)	Quick DASH score 18±10

NR: Not reported.

### Statistical analysis

The population size and the mean and standard deviation values for EVA pre-intervention and post-intervention, as well as the ASES score pre-intervention and post-intervention, were subjected to meta-analysis, and the results were expressed as mean differences. The percentage of variability attributable to heterogeneity among the studies was estimated using the I² statistic, with a p-value of less than 0.05 considered statistically significant.

Heterogeneity was classified based on I² values as follows: 25% low heterogeneity, 50% moderate heterogeneity, and 90% high heterogeneity. A random effects model was used, and meta-analyses were performed with a 95% confidence interval (CI).

All statistical analyses were performed using the Cochrane Revman software.

Table 3 shows that there was a difference in the average ASES function before and after the intervention, indicating an improvement in function after treatment. Similarly, Table 4 also shows that there was a difference in the average pain levels before and after the intervention, indicating a reduction in pain after treatment.

**Table 3:** Statistical analysis of pre and post-operative ASES score values of lower trapezius transfer.

		Pre ASES			Post ASES			Avarege difference	Avarege difference
Study or Subgroup		Significant	SD	Total	Significant	SD	Total	IV, Aleatory, IC95%	IV, Aleatory, IC95%
Almeida et al., 2023^17^		26.63	7.34	10	75.24	3.59	10	23.3% −48.61 [−53.67, −43.55]	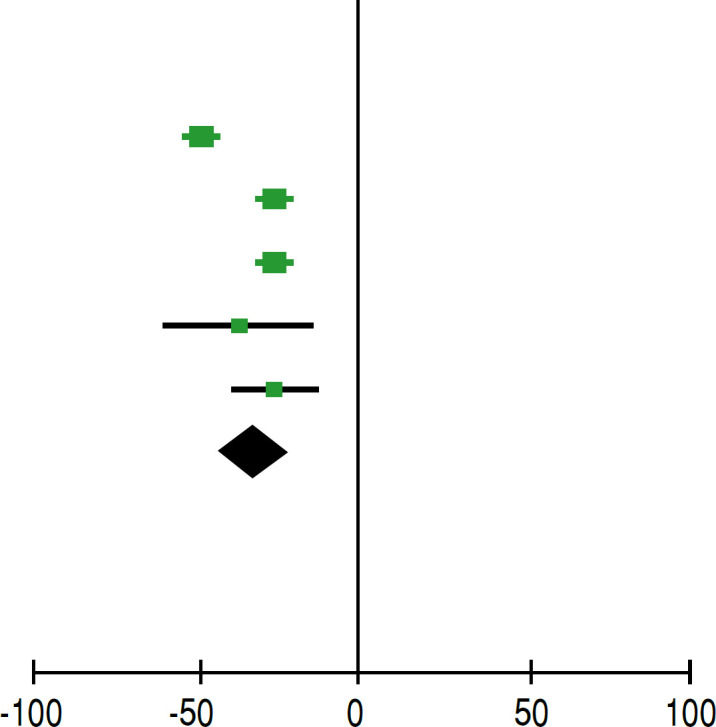
Baek et al., 2022^18^		57.1	13.4	36	83.7	8.2	36	23.2%-26.60 [-31.73,-21.47]	
Bael et al., 2022^19^		55.8	16.7	42	82.7	8.6	42	23.0%-26.90 [−32.58, −21.22]	
Baek et al., 2023^20^		47.2	17.3	36	84.8	67.6	36	12.3% −37.60 [−60.39, −14.81]	
**Subtotal (95% CI)**				**124**			**124**		
	Heterogeneity: Tau² = 138.61; Chi² = 47.17, df = 4 (P <0.00001); I² = 92%								
	Test for overall effect: Z = 5.70 (P<0.00001)								
								
Test for subgroup differences: Not Applicable									
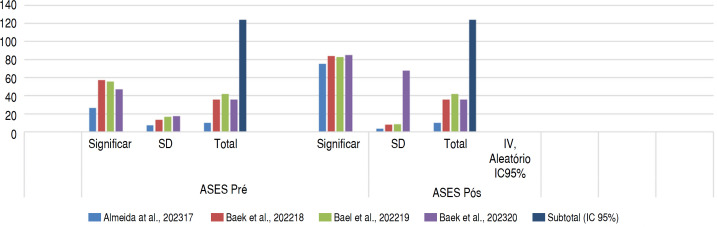								

**Table 4:** Statistical analysis of pre and post-operative VAS values of lower trapezius transfer.

		Pre VAS			Post VAS				Avarege difference	Avarege difference
Study or Subgroup		Significant	SD	Total	Significant	SD	Total	Weight	IV, aleatory, IC 95%	IV, aleatory, IC 95%
Almeida et al., 2023^17^		7.9	0.74	10	2.2	2.2	10	13.20%	5.70 [4.26, 7.14]	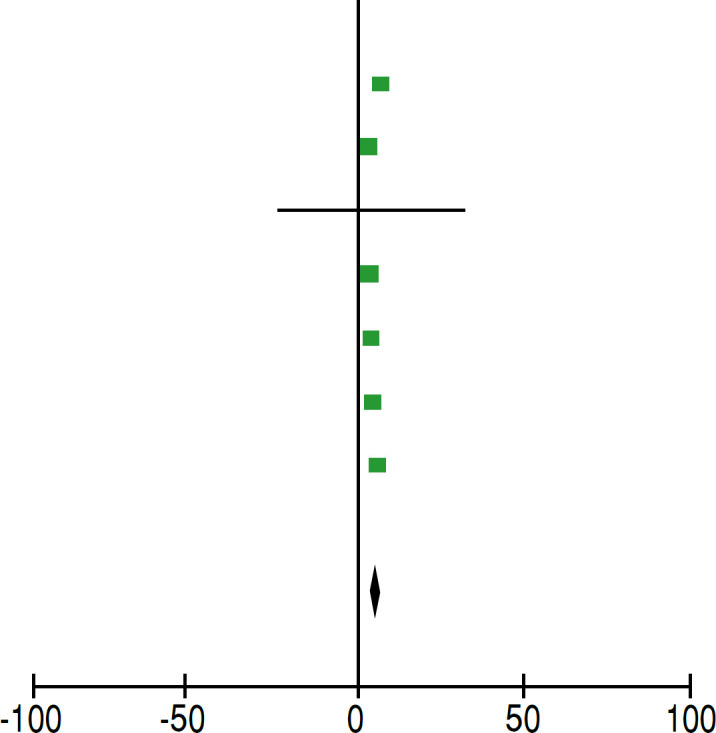
Baek et al., 2022^18^		4.3	16	42	1.7	1,3	42	18.30%	2.6 [1.98, 3.22]	
Baek et al., 2022^19^		4.5	61.8	36	1.3	61	36	0.10%	3.20 [-25.17, 31.57]	
Baek et al., 2023^20^		4.5	1.2	36	1.4	0,7	36	19.10%	3.10 [2.65 3.55]	
Bozoglan et al., 2023^21^		6.1	1.1	16	2.4	1,35	16	17.00%	3.70 [2.85, 4.55]	
Marigi et al., 2023^22^		6,2	2.2	72	1.1	2	72	18,00%	5.10 [4.41, 5.79]	
								
**Total (95% CI)**				**212**			**212**			
	Heterogeneity: Tau² = 1.04; Chi² = 41.66, df = 6 (P < 0.00001); I² = 86%									
	Test for overall effect: Z = 8.66 (P < 0.00001)									
Test for subgroup differences: Not applicable										
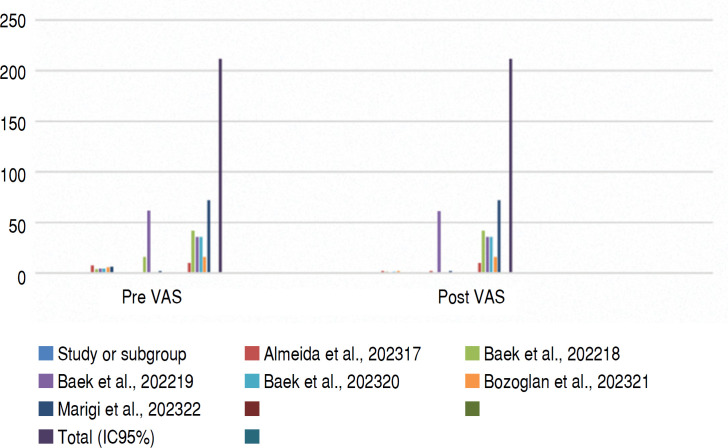									

## DISCUSSION

This review included 12 studies,^17–28^ compiling a total of 374 whose results showed a significant reduction in pain and improvement in joint mobility of the affected shoulders after LTT surgery, the subject of this study.

Initially, the first studies conducted using this technique employed experimental designs on the hemithorax of fresh-frozen cadavers. These studies mainly investigated the biomechanics of this intervention to assess the range of motion of the shoulder joint. This provides a greater restoration of external shoulder rotation compared to the transfer of the latissimus dorsi.^30^ Moreover, the LTT determines a significant decrease in superior and anteroposterior translation. It also causes a peak of subacromial contact pressure at 0°, 30°, and 60° of shoulder abduction. The LTT efficiently prevents the loss of abduction movement and the superior migration angle of the humeral head. Therefore, we agree with the authors who argue that the LTT can sufficiently restore glenohumeral kinematics, potentially leading to better postoperative functional outcomes.

In 2016, a study conducted on 32 patients demonstrated that, after an average follow-up of 47 months, there was a significant improvement in pain, function, and range of motion of the shoulder. In patients with more than 60° of preoperative flexion, the gain in shoulder range of motion was significantly greater. External rotation improved in all patients, regardless of the loss of extension observed in the preoperative period.^24^

Overall, studies have shown good clinical and surgical results.^17–28^ In patients with a notable reduction in rotation strength, tendon transfers have the ability to restore strength while safely preserving the joint.^22^ After surgery, increases in forward flexion and external rotation are expected, as well as a reversal of the external rotation delay signal when present before surgery.^8^

We observe that with the inclusion of more studies on this topic, the surgical technique of LTT has undergone significant improvement. This has been used assistively with the aid of arthroscopy and is considered a promising option for the treatment of this condition.^18–20,22–26,28^

It is important to highlight that the transfer of the lower trapezius tendon should not be used in certain patients such as: those with arthropathy due to rotator cuff tear; combined loss of elevation and external rotation; irreparable tear of the subscapularis muscle; involvement of the teres minor muscle; and in elderly patients or those considered unable to comply with the strict rehabilitation guidelines.^6^

We highlight the sample value and observed results as relevant positive aspects of this secondary study. We cannot fail to point out some negative aspects of this systematic review where we highlight the low level of evidence of the compiled studies, with most being retrospective cohort studies. The homogeneity among the studies was not evaluated. Authors who used combinations of different surgical techniques, approaches (open versus arthroscopic), and types of grafts were excluded. Some studies presented biases when considering sample size and minimum follow-up time, which hindered the identification of more relevant considerations.

Thus, we emphasize that this interesting topic requires the pursuit of scientific work with the highest level of scientific evidence that can be achieved through randomized clinical trials and the performance of representative sample calculations.

## CONCLUSION

The authors conclude that the results of the pre and postoperative evaluation of patients with irreparable rotator cuff injuries treated by the LTT technique showed:

Improvement of pain after the surgical intervention was performed. The average pain intensity was 7.1 to 2.4, as measured by the VAS. Improvement in function, as measured by the ASES system. The initial average value was 49.5 (a poor result) and subsequently improved to 78.3 (a good result).

The associated p-value indicated a statistically significant difference in pain levels before and after the intervention. The transfer of the lower trapezius is an effective therapeutic option for patients treated with the studied surgical technique, according to the results obtained.
